# The Often-Unmentioned Key Points of Orthotics—A Short Comment

**DOI:** 10.3390/children12091154

**Published:** 2025-08-29

**Authors:** Reinald Brunner

**Affiliations:** Department of Biomedical Engineering, University of Basel, Hegenheimermattweg 167B/C, 4123 Allschwil, Switzerland; reinald-g-h.brunner@unibas.ch

**Keywords:** posture stability, orthotics, gait disorders, neuromuscular diseases, biomechanics, coronal and sagittal plane stability

## Abstract

Orthoses are a vital part of treating gait disorders, especially in children and adolescents with neurological and neuromuscular conditions. For proper walking, the supporting leg must be stable to allow the other leg to swing forward and take a step. Stability is also essential for motor development. This stability depends on the inclination of the tibia, which needs to be kept upright during mid-stance in both the sagittal and coronal planes. Controlling the load axis in all planes and the foot in the transverse plane helps maintain proper tibial control. More studies are now examining the effects different orthoses and designs. While much focus has been on the sagittal plane, there is much less information about how orthoses influence the coronal plane or foot control. As a result, there is limited guidance from the existing literature. Children who find it hard to express discomfort or negative effects may simply reject orthoses altogether. This paper explains how important proper tibia inclination and control on the load axis are in all planes and how they affect stability. The foot acts as a lever for the gastrosoleus muscle, which controls the tibia. In case of foot instability or deformity, the foot requires support that takes into account the changing load when walking. I also emphasize that these points are regularly considered when studies are reported.

## 1. Introduction

Orthoses have been used for hundreds of years to correct deformities and treat functional deficits. In the early days of orthopaedics, they were of great importance as there were no or only limited surgical and anaesthesiologic techniques. Today, orthopaedic treatment has shifted to surgery, and the importance and knowledge of conservative orthopaedic treatment is declining. Nevertheless, the number of publications on orthoses has almost tripled every 20 years and has now reached around 1000 studies per year. Around a third of related scientific studies deal with functional orthotics and about 80 concentrate on the effect in children.

Functional orthoses are very important for patients with neurological and neuromuscular disorders. Children learn their basic motor skills in the first few years of life. Paralysis or tone disorders make normal functioning difficult. The sensory system provides important information about the position and movement of the body parts, joints, and muscles. This information is required to send the right signals to move properly. If this control is impeded, it can cause additional problems already in early childhood. Motor and sensory disorders lead to delayed or incomplete and faulty development. This is common in children with cerebral palsy, who have a similarly strong affection of both the motor and the sensory tracts in the brain [1]. Muscle activity differs from normal. There is some debate about whether muscle tightness is spasticity or compensation [2]. As a result, deformities can develop, such as shortened muscles or bone deformities due to abnormal use. Children may also struggle mentally because of these movement problems. They might have trouble balancing, feel insecure, and lose motivation to move or play. Their confidence in their own body may be limited. Psychological effects are hard to measure but are very important. Especially in the early years, when aggressive treatments have little place, orthotic treatment is an essential treatment modality. The aim is not only to prevent deformities but also to give stability and help children feel more confident in their bodies. To do this, orthoses guide movements and help correct how children move.

One superior goal is to improve functional disorders, such as gait disorders, by compensating for functional and correcting structural deformities. Orthoses can sometimes be the only option or a temporarily alternative to surgery. The success of a functional orthosis heavily depends on its biomechanical impact. Usually, the design is customized to meet the individual’s specific needs and problems. While functional deformities can often be corrected, structural deformities are virtually inaccessible. In spite of all the deformities and problems to fit orthoses, the goal is to improve function, and dynamic stability is crucial. Surprisingly little, however, has been written in the literature about the factors in an orthosis that cause dynamic instability during function. If an orthosis has design flaws from a biomechanical perspective, a child may struggle with it or reject it altogether. Children often cannot communicate negative effects or discomfort as clearly as adults do, making the biomechanical details especially important for them. Since children and adolescents are still growing, their orthoses and types are frequently changed, so providing optimal support with every new orthosis is crucial.

Many construction details determine whether an orthosis successfully improves function or causes additional problems. The higher an orthosis extends up the leg, the greater is the impact on tibial inclination and hence dynamic stability. I want to emphasize three key points: controlling tibial inclination, keeping the load axis in the support area, and foot control.

## 2. Biomechanical Considerations for Stability

There are multiple biomechanical effects of orthoses, and some have been topics of publications. These considerations apply equally to children and adults. I will concentrate on the effects on stability during stance and the stance phase of gait. Gait disorders in neurological and neuromuscular diseases primarily result from the problem of maintaining stable postural control during movement. In some of these diseases, the reason for this issue is the disruption of the sensory systems, but functional and structural deformities always increase the difficulty of achieving balance and body control. Firstly, I describe the prerequisites for a normal, functional and stable gait, and then the design requirements that orthoses must fulfil to achieve a gait that is as stable as possible and as close to the norm in the case of deformities.

**Figure 1:**
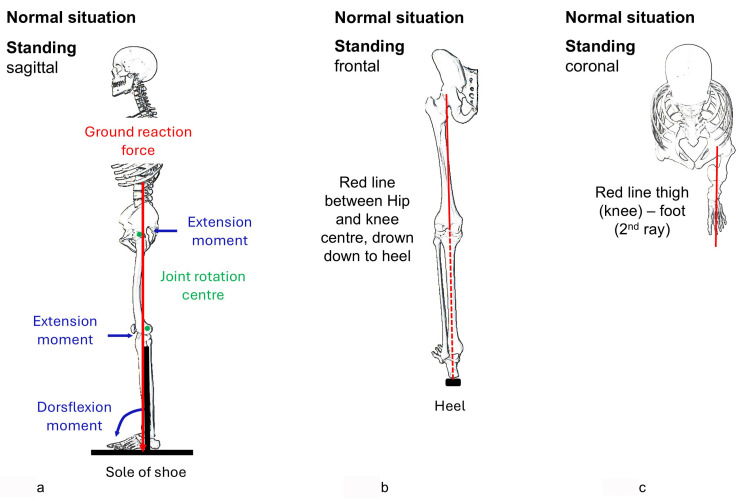
(**a**–**c**) Schematic drawing of axes of load: in the sagittal plane, the ground reaction force (red) falls just behind the hip joint centre, just in front of the knee joint centre and through the midfoot area, the tibia is vertical (**a**); In the coronal plane, the load axis—defined as the extension of the line between the hip and knee joint centers (red)—passes through the middle of the heel, which forms the support area (**b**); and in the transversal plane, the foot is in the direction if the leg (clinically the axis hip-knee, red) (**c**). Deviations lead to instability in the respective plane.

To stand in a stable posture, the centre of mass must be above the support surface that contains the starting point of the vertical ground reaction force. For optimal posture with little need for control and muscle activity, the feet should be facing the direction of the leg and function, the foot should touch the ground from heel to toe, and the knee and hip joint should be only slightly hyperextended to create an external extension moment (Figure 1a).

Control of the tibia is crucial. Any deviation from plumb in the sagittal plane requires compensatory positions at the knee and hip levels. If the tibia is tilted forward the knee and the hip need to be flexed in order to keep balance and the ground reaction force in the support area. This crouched posture requires increased muscle activity and body control. A stable foot in the direction of activity acts as lever for the gastrosoleus muscle which actively controls the tibia in the sagittal plane. There is, however, no muscle to control the coronal plane position. The rearfoot deviates into valgus or varus in foot deformities. This shifts the support area at the heel. The ground reaction force, acting between the foot and the ground, always lies within the loading area and changes the angle of incidence in case of deformities. Concerning stability in the coronal plane, these data, as well as the kinetic data from gait analysis, are difficult to interpret. In the sagittal plane, compensations can be achieved through appropriate joint positions, but this is not possible in the coronal plane. For assessment, the extension of the line from the hip joint to the knee joint onto the ground helps, called the load axis. In normal legs, the tibia must be vertical, and the load axis must be in the middle of the support area (Figure 1b). The foot points in the direction of the leg (Figure 1c). Besides this clinical rule of thumb, instrumented 3D gait analysis offers the possibility of dynamic examination. However, the relevant angles (tibia to floor, foot to shank) are often not shown.

Walking requires stability on only one leg. The support surface is smaller, but it still contains the ground reaction force, which now deviates in three dimensions and travels along the foot. This means that the ground reaction force is no longer necessarily vertical and other factors such as acceleration and inertia contribute to dynamic stability. During normal walking, there is contact first only with the heel, then with the whole foot, and at the end of stance phase only with the toes. For an orthotic fitting, this means that first the heel, then the midfoot, and finally (at push-off) the forefoot including the toes need to be controlled by a respective imbedding to control the foot at any time. Control of the heel is best achieved with a varus heel pad in valgus deformities (the reverse in varus deformities). The midfoot arch is supported along its full length to prevent arch collapse. Longstanding valgus or varus deformities both lead to fixed forefoot supination, which requires support of the first ray to prevent the supination deformity from pushing the forefoot out of the axis.

As a rule of thumb, the situation in mid-stance is close to that of standing on one leg. To be stable in this situation, the tibia must be vertical in the sagittal and the coronal plane, and the load axis must be supported. Only a vertical tibia makes it possible to keep the knee and hip extended, a position in which external extensor moments and internal ligamentous structures take over control. This ensures intrinsic stability. The foot facing the direction of action helps with achieving tibial control and improves stability. Whilst in the sagittal plane, muscle control and compensatory joint positions in the leg exist as another option to regain stability, this is not the case in the coronal plane. A varus or valgus tibia leads to instability. The load axis connects the hip and knee joint centres and extends this line to the floor. Poor placement, such as a valgus or varus foot, shifts the loading area to the side and leads to inward or outward collapse. In both cases, trunk movements to the side are necessary to regain stability.

**Figure 2:**
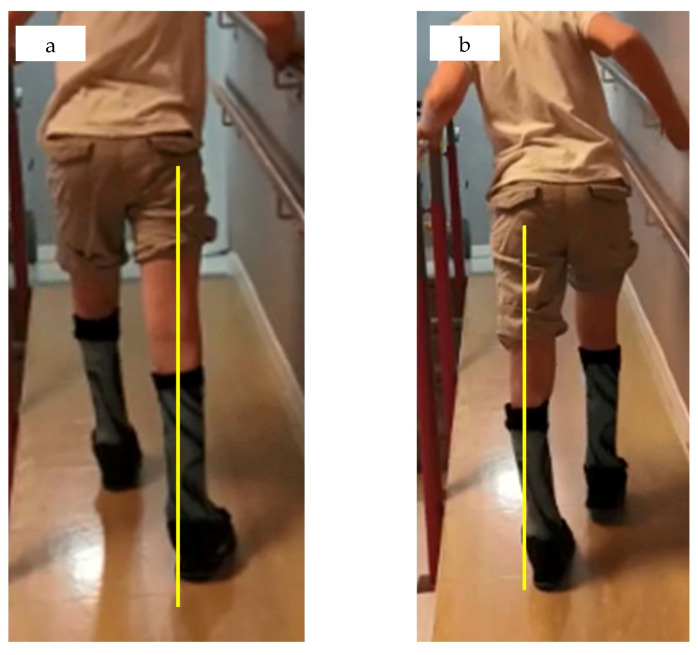
(**a**,**b**) Example of coronal plane instability. The load axis is drawn in yellow. (**a**) The AFO is too valgus in the heel and the leg collapses inwards with the tibia and the knee into valgus. (**b**) opposite situation with an AFO in varus. In addition to lack of stability, the right foot has a tendency to external, the left to internal rotation.

Orthoses should, therefore, keep the tibia plumb, ensure full foot–ground contact. Weakness or dysfunction of the gastrosoleus muscle leads to missing control on tibial inclination in the sagittal plane. The orthosis must restore this control. However, the effect of the plantar flexors or an orthosis on tibial control to extend the knee also depends on the position of the leg segments in space. This mechanism may lose its effect with knee flexion of over 30 degrees [3]. In the coronal plane the load axis needs to be supported by extension of the heel to the side. In the coronal plane, the leg often collapses in valgus combined with external rotation or, more rarely, in varus with internal rotation (Figure 1b and Figure 2a,b).

Gait analysis relies on the intersection of planes that represent the body segments in three-dimensional space. These planes are defined by markers positioned on the segments of the legs. The angles projected onto the standard planes (sagittal, coronal, and transverse) are calculated. Together with the ground reaction force, inverse dynamics are used to compute the net joint moments and mechanical powers at the various joint levels. This data enables inferences about muscle activity, which is typically corroborated by dynamic electromyography (EMG). While this method is standard for the sagittal plane, understanding the situation in the other planes is often challenging, if not impossible. Since the coronal plane analysis uses the ground reaction force rather than the load axis, conclusions regarding necessary orthosis adaptations are less obvious. In the transverse plane, the foot rotation angle—defined as the angle between the foot and shank segments—describes rotation rather than the foot progression angle, which reflects the foot’s orientation relative to the direction of gait.

Poor setup of any orthosis may lead to a perception of instability. Standing and walking becomes impaired, activity is reduced, and more demanding activities such as running or jumping are avoided. In children, motor development is retarded. In order not to lose balance, the patient increases the muscle tone. The perception of instability may be one reason for a tone rise which may even be regarded as spasticity in patients with brain affections. Designing orthoses for children and adults with gait disorders is not just a technical process. It impacts their quality of life and development outcome profoundly.

## 3. Construction of the Orthosis Regarding Stability of Posture


**The issue with joint angles**

Angles are employed to characterize segmental positions of the body and to describe movement, yet these measures have certain limitations.

Sagittal plane:

Thigh axis: midline of sagittal projection. (The femur, the principal loaded structure, is anatomically situated at the center of the segment.)

Shank axis: The loaded element, the tibia, is positioned eccentrically within the segment. There is no anteromedial muscle, while the greatest muscle mass is dorsolateral.

- The anterior tibial border represents the tibia.

- The segment’s midline depends on muscle mass; the stronger the muscles, the more this line deviates from the anterior border (more forward tibia lean).

- Posterior border (from cast mold): depends even more on muscle mass.

Foot: lateral inferior border

- “Normal” position. Difficult to maintain in unstable and deformed feet during measurement, especially if there are additional muscle contractures (the deformity masks the contracture).

- Foot in full supination. Locking the hindfoot compensates for instability, making muscle contractures more reliable to assess.

- Foot left completely free in its deformity. The foot deformity masks the muscle contractures.

The various axis combinations used for the knee and ankle yield different angles. In my experience, the anterior border of the shank with the foot in supination has proven most reliable. The difference between the supinated and free-foot position reflects the amount of dorsiflexion from foot instability that cannot be controlled by the plantar flexors.

With an orthosis, measurement conditions differ. For the tibia, the issues are the same.

- Orthosis alone. The orthosis corrects and holds the foot. The inferior lateral border of the orthosis defines the foot axis. 

- Functionally: Sole of shoe (equivalent to floor. The tibia should be held vertical to the floor.) 

Coronal plane:

Varus/valgus at knee and calcaneus do not indicate whether the extended axis hip-knee joint center (loading axis) passes through the support area.

Transversal plane:

Tibial rotation: femur against foot. The femoral condyles or the patella; or when the knee is flexed, the thigh axis. Foot: The axis of the first intermetatarsal space or the second ray. 

For this paper, the axes employed are those that most closely reflect the biomechanical condition:

Sagittal plane:
-
Midline of the thigh
-
Anterior boarder of the shank
-
Lateral inferior boarder of the sole of the shoe

Coronal plane: 
-
Load axis (extension of the line connecting hip and knee center)

Transversal plane:
-
Thigh midline
-
Second toe (estimated if not visible in the orthosis)



**Figure 3:**
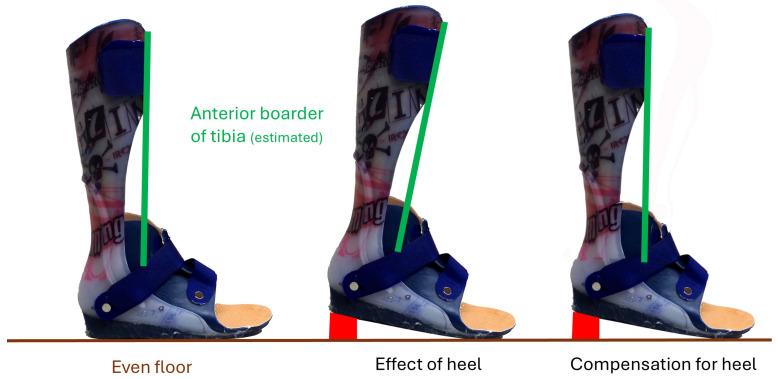
Shows the effect of a heel with a 90 degrees AFO on the inclination of the tibia and the increase of plantar flexion (equinus) position to compensate.

Functional orthoses are greatly important, but descriptions of design are often restricted to providing minimal and general information and details concerning the effect on dynamic stability and the position of the tibia and load axis are missing. At best, they cover the sagittal plane. This applies to both children and adults. For example, ‘The foot is positioned at an angle of 90 degrees’; such a description seems clear, but it is relevant which axis is taken for the shank (the midline, the posterior border, or the anterior border of the shin, see boxed text “the issue with angles”) and whether the 90 degrees in question is measured with or without the shoe and its heel. Most shoes have a heel and therefore push the shin into a flexed position while the foot is held at 90 degrees by the orthosis. A little equinus would be required to compensate for the heel (Figure 3). The description ‘The foot is positioned at an angle of 90 degrees’ is, thus, not clear, as there are regional differences in the use of axes and in construction standards, as well as in certain specialised centres and in the learning content in the training of orthosis manufacturers. The relevant sagittal angle with an orthosis is the angle between the axis of the tibia (best anterior boarder) and the lateral boarder of the shoe. The other planes, coronal and transversal, are usually not mentioned. The literature search (Pub Med) for “orthosis” AND “tibial inclination” gave 0 results, the same for “orthosis” AND “tibial inclination”. The search for “orthosis” AND “tibial position” gave 2 results, but these were studies only of foot pads and foot orthoses. “Load axis” AND “gait” showed two papers but with osteoarthritis or tumour, and again both were not considered. “Load axis” AND “orthosis” gave 1 study on the load axis in relation to the knee joint centre in osteoarthritic knees. “Ground reaction force” AND “coronal” AND “orthosis” showed 4 papers. Out of these 4, two studied effects of higher orthosis (ankle foot orthoses, AFO). Both papers report moments in all planes but do not address the localisation or position of the load axis in respect of the contact area nor do they describe how the foot was held [4,5]. Although tibial inclination and the position of the load axis in the sagittal and coronal plane are crucial factors for functional stability, there is no literature in this regard especially for higher reaching orthoses.

Poor support in the coronal plane results in instability to the side. This may be caused by a tilt of the tibia to the side or a lack of support under the load axis. Feet with poor muscle control tend to deform under load which results in a rotational abnormality and shifts the loading area to the side (Figure 2a,b). If the load axis is not within the support area, the posture is unstable. Valgus occurs when the load axis falls on the medial portion of the contact surface, or even more medially. The coronal-plane posture is unstable. Additionally, the rocking of the foot during gait can cause external rotation of the foot, which may increase during the stance phase. A similar pattern occurs in the opposite direction with varus. Increased malrotation reduces the effectiveness of plantar flexor control on the tibia, causing the leg to collapse into flexion. When viewed from the front or back, the leg axis may appear valgus or varus. It is important to position the heel so that the load axis is centred within the support area. If the line falls outside this area, the heel can be shifted to the appropriate side or extended as needed. The same biomechanical considerations are actually made when prostheses are built. The load axis is crucial. Many orthopaedic technicians use special instruments for a correct setup, such as the 3D L.A.S.A.R. Posture (Ottobock).

As noted earlier, the standard 3D gait analysis available today provides limited guidance on how to adapt the orthosis, and this analysis is time- and resource-intensive. Innovative developments in gait analysis are moving away from markers, which may make the analysis much easier. New software, potentially incorporating AI, could identify coronal-plane problems more effectively than the load axis alone, with even clear indications how to change an orthosis.

The foot acts as a lever for the gastrosoleus muscle and as support area for the load axis. The foot imbedding in the orthosis stabilizes the foot, corrects the heel deformity, and aligns the foot in the desired direction as described earlier. This embedding must follow the moving load vector and control the foot during the three rockers during the stance phase of gait. In the case of gastrosoleus contracture, part of the dorsiflexion occurs at the hindfoot and midfoot. The motion axis is oblique, resulting in a combination of dorsiflexion with external rotation and flattening of the foot arch. Severe forms are referred to as a midfoot break. The deformity can still be flexible, allowing the foot to be positioned anatomically but only in an equinus position at the ankle. When the foot is supple enough, as is often the case in children, holding it in the anatomical position unmasks the gastrosoleus contracture. If 90 degrees of ankle dorsiflexion are enforced, the foot deviates further in its deformity. The dilemma for the construction of an orthosis is whether to correct the foot anatomically, keep it aligned in the transversal plane, and compensate for the resulting equinus contracture with a heel, or to leave the foot valgus external rotation deformity uncorrected and set the ankle at 90 degrees. To the author’s knowledge, there is no description in any publication in which such a choice has been made, nor is there a generally accepted standard for treatment. However, if the heel is allowed to deviate into valgus, the Achilles tendon progressively shortens especially in the growing child.

**Figure 4:**
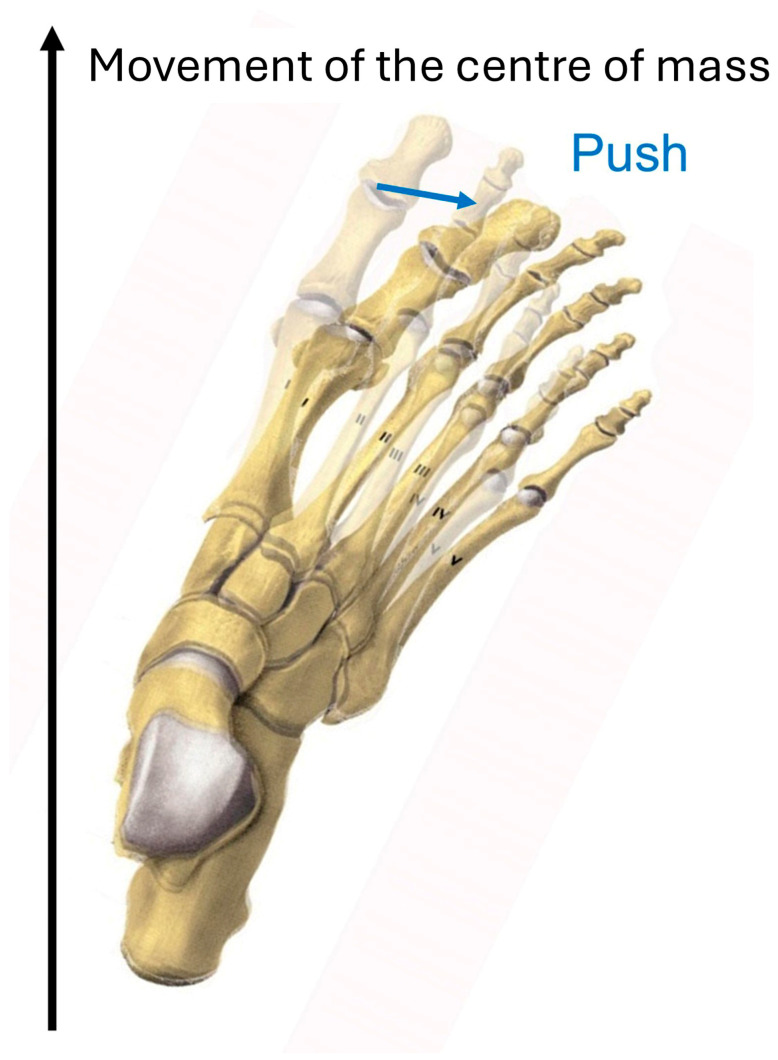
Rocking over a malrotated foot increases the rotation as the midfoot and toes are pushed to the side when the load progresses.

The foot must be maintained in the direction of gait and the leg (Figure 1c). A flexible foot deformity, common in children and facilitating corrections in this population, can be corrected with an orthosis. With more rigid deformities, this correction is more difficult, but adjustments in the heel and sole area, usually extensions, can at least partially compensate for persistent malrotation. If the foot remains in a rotational malposition, the forefoot and toes are pushed to the side whilst rocking over the foot (Figure 4). Such a problem also happens with an orthosis which does not correct the foot deformity in the transversal plane.

Finally, the shoes also influence stability. A heel in combination with an AFO at 90 degrees leads to a forward tilt of the tibia and knee and hip flexion as a consequence (Figure 3). The normal posture (Figure 1a) allows a person to stand with minimal energy and easy balance control. Any deviation from this posture is perceived to be more demanding and generally avoided. If, however, the orthosis does not include the shank, the patient is free to position the tibia, but this freedom is only beneficial if the patient can control it.

The orthosis only covers the functional deficits of the patient’s leg control. If the patient can control the position of the tibia, the orthosis can be low and leave the shank free. In the case of a short gastrosoleus muscle/Achilles tendon, the contracture can be used to control the tibia, and the orthosis only needs to prevent plantar flexion. These examples show that the design of the orthosis is tailored to the patient’s needs. With the construction of a rigid or resilient orthoses without a joint, the remaining elasticity is a relevant factor for the function of an orthosis.

## 4. Description of Orthoses

I have explained the direct effects of orthoses on posture control and stability. The higher they reach up the leg, the more they determine the position of the segments of the leg, rather than the patient. This is especially important for the growing and developing child with neuromuscular disorders such as cerebral palsy where motor control is impaired. If the effects of orthoses are studied or discussed, it is crucial that these details regarding the construction and the effects on the foot, load axis, and tibia are well described. However, such construction details are rarely mentioned. Small errors can turn a well-functioning design into a non-functioning orthosis that even impairs the patient’s function. These details are not always taken into account. For example, a comparison of low supramalleolar and ankle–foot orthoses in 1999 concluded that AFOs are less well tolerated. The illustration in the paper shows a tibial forward tilt with the AFO but not with the other type of orthosis [6]. It is no wonder that the patients had difficulty standing and rejected the higher orthoses. The effect in the coronal plane is of similar importance. A negative effect can be so strong that it prevents the patient from walking independently. Finally, the rotational position of the foot in relation to the shank or leg is important for postural control. As already explained, it is possible, especially in children, to position the foot in different directions before structural deformities develop. It is surprising that information available about these important factors is only rudimentary, if it is available at all. Without this information, however, it is difficult or even impossible to understand the results and conclusions of scientific papers or clinical reports. Medical practitioners find little help with difficulties when they consult the literature.

In older and even current literature, essential information required to comprehend the effects of an orthosis is often poor, incomplete, or missing. Specifically:

- the tibial position and how it is controlled by the orthosis in conjunction with the respective shoe;
- coronal plane instability and collapse of the leg due to inadequate support of the load axis;
- how the foot is controlled, especially in the presence of deformity and muscle contractures, including the dilemma between a 90-degree at the ankle with remaining foot deformity and equinus with the foot aligned.

## 5. Conclusions

In children and adolescents, orthotic treatment is an essential pillar of therapy. The same type of orthosis can be beneficial or harmful depending on its design. Both for clinical discussions and in publications, the details of the orthosis design must be made clear so that the lack of effect and positive or negative effects can be understood. I stressed three points crucial for stability:

- The tibia must be vertical
- The load axis must fall into the support area (in the centre while standing)
- The foot must point in the direction of the leg
